# The Complement System in Metabolic-Associated Kidney Diseases

**DOI:** 10.3389/fimmu.2022.902063

**Published:** 2022-07-18

**Authors:** Ziyu Xu, Li Tao, Hua Su

**Affiliations:** Department of Nephrology, Union Hospital, Tongji Medical College, Huazhong University of Science and Technology, Wuhan, China

**Keywords:** complement system, kidney diseases, metabolic syndrome, innate immunity, metaflammation

## Abstract

Metabolic syndrome (MS) is a group of clinical abnormalities characterized by central or abdominal obesity, hypertension, hyperuricemia, and metabolic disorders of glucose or lipid. Currently, the prevalence of MS is estimated about 25% in general population and is progressively increasing, which has become a challenging public health burden. Long-term metabolic disorders can activate the immune system and trigger a low-grade chronic inflammation named “metaflammation.” As an important organ involved in metabolism, the kidney is inevitably attacked by immunity disequilibrium and “metaflammation.” Recently, accumulating studies have suggested that the complement system, the most important and fundamental component of innate immune responses, is actively involved in the development of metabolic kidney diseases. In this review, we updated and summarized the different pathways through which the complement system is activated in a series of metabolic disturbances and the mechanisms on how complement mediate immune cell activation and infiltration, renal parenchymal cell damage, and the deterioration of renal function provide potential new biomarkers and therapeutic options for metabolic kidney diseases.

## Introduction

### Metabolic Syndrome

Metabolic syndrome (MS) is a group of clinical abnormalities, which is characterized by central or abdominal obesity, hypertension, hyperuricemia, and metabolic disorders of glucose or lipid (1). According to the Center of Disease Control and Prevention (CDC), the United States witnessed an increase in the prevalence of metabolic syndrome by more than 35% from 1988 to 1994 and 2007 to 2012 (2). Additionally, as a disease previously thought to be associated with western lifestyle and habits, the incidence of metabolic syndrome is now on the rise in developing countries, leading to an estimated prevalence of 25% in the general population (3), which has become a challenging public health burden. Currently, the etiology of MS is still unclear; in addition to genetic and epigenetic factors, overnutrition and sedentary lifestyles are usually thought to be related to its occurrence. Besides abdominal obesity and insulin resistance (IR), the core manifestations of the syndrome (4), MS is also considered to be an important risk factor for multiple diseases, such as cardiovascular disease (CVD), type 2 diabetes mellitus, chronic kidney disease (CKD), arthritis, and even several types of cancer (5–7).

As an important organ involved in metabolism, the kidney is inevitably attacked by various metabolic abnormalities. It has been suggested that individuals with MS has an increased risk for developing renal damage, clinically expressed in the form of microalbuminuria and/or chronic renal dysfunction (8). According to a study based on a large, representative sample of the U.S. general population, metabolic syndrome is a strong and independent risk factor for chronic kidney disease. In addition, the more abnormal metabolic components are, the higher is the risk of renal impairment (9). After excluding the effects of glycemic and blood pressure, MS remained an independent risk factor contributing to the development of CKD (10). Among patients receiving kidney transplants, those with pre-transplant metabolic syndrome have an increased probability of *de novo* post-transplantation diabetes mellitus (PTDM), chronic graft dysfunction, and even graft loss (11, 12). Compared with controls, kidney lesions in patients with metabolic syndrome are characterized by tubularatrophy and interstitial fibrosis, as well as described as microvascular and obesity-related glomerular changes (13).

Therefore, exploring the pathogenesis of metabolism-related kidney diseases is an important part of the prevention and treatment of CKD. It has been reported that inflammation, insulin resistance, inappropriate activationof the renin–angiotensin–aldosterone system (RAAS) are involved in the progression of metabolism-related kidney diseases, while the association between complement system and these diseases has not been reviewed. In this review, we updated and summarized the mechanisms on how the complement system causes renal damage in different metabolic disorders and discussed possible biomarkers and potential therapeutic targets.

### Complement System and Kidney Diseases

**Figure 1:**
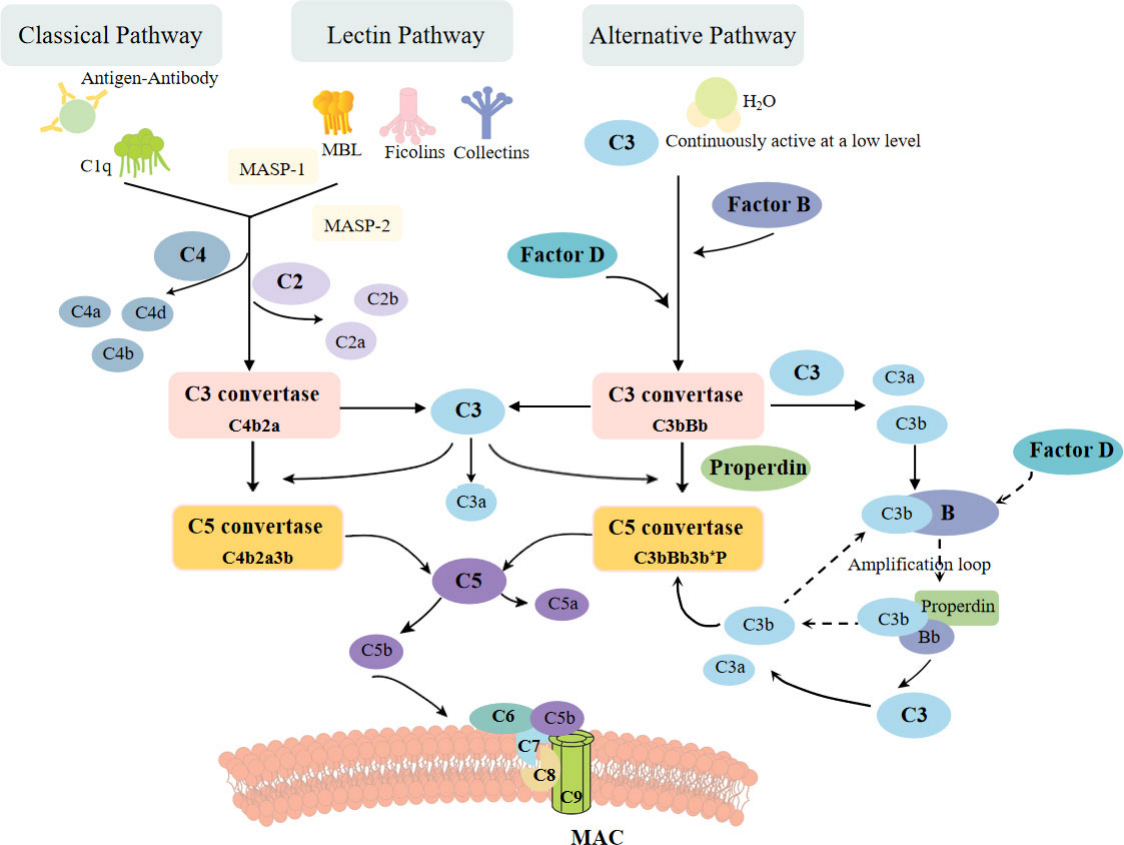
The complement activation pathways: classical, alternative, and lectin pathway. The classic pathway is triggered by binding of antigen–antibody complexes to C1q. The lectin pathway begins with signal recognition by oligomeric structures of MBL, ficolins, and collectins, which active MASP1 and MASP2, thus in turn mediate the production of C4b. Both pathways then lead to the formation of common C3 convertase, C4b2a complex. In the alternative pathway, a small fraction of the C3 molecules is hydrolyzed, exposing new binding sites and then combined with factor B protease. After cleaved by factor D, another C3convertase (C3bBb) is formed, leading to cleavage of further C3, and this process is perpetuated through an amplification loop. All three pathways ultimately result in the cleavage of C3 and C5, leading to the formation of MAC, which inserts into membrane and then induces cell lysis.

The complement system, a fundamental component of innate immune responses, was traditionally considered as the “first line of defense” against microbial intruders (**Figure 1**). Today, the complement system is recognized as a connecting link between innate and acquired immunity, participating in various processes, such as synapse maturation, clearance of immune complexes, angiogenesis, tissue regeneration, and lipid metabolism (14). Studies have shown that the activation of complement system is implicated in various kidney diseases. In non-immune complex-mediated kidney diseases, uncontrolled complement activation is the primary driver of atypical hemolytic uremic syndrome (aHUS) and C3 glomerulopathy. And in those immune complex-mediated kidney diseases, complement also plays a prominent role in anti-glomerular basement membrane (GBM) disease, lupus nephritis, membranous nephropathy, and IgA nephropathy. Additionally, after renal transplantation, abnormal activation of complement in ischemia-reperfusion and antibody-mediated rejection (ABMR) may lead to inflammation and graft dysfunction (15, 16). Therefore, therapeutic agents which target complement pathways are essential and urgent for these diseases. Eculizumab, a monoclonal anti-C5 antibody, has been proven effective in the treatment of aHUS (17–19), reducing complement activation, endothelial damage, thrombosis, and inflammation, improving renal function in adult patients. In patients with C3 glomerulopathy, the results of eculizumab were mixed (20, 21), requiring more rigorous and multicentric clinical trials. Furthermore, new anti-complement drugs are on the way. Inhibitors of C5a receptor1 (C5aR1), C3, factor B (FB), and factor D (FD), as well as an anti-MBL-associated serine protease 2 (MASP2) monoclonal antibody are under investigation (22). In IgA nephropathy and lupus nephritis, clinical trials which targets MASP2 and C3 are also ongoing (23). Inhibitors of C3 and C5 convertases, together with drugs that target the classical and lectin pathways of the complement system, are highly prospected to improve graft function after transplantation (15). Therefore, exploring the mechanisms on how the complement system mediates renal damage in different metabolic disorders will provide new options for the treatment of metabolic kidney diseases.

## Complement System in Metabolic-Associated Kidney Diseases

### Diabetic Kidney Disease

Diabetes mellitus (DM), a group of metabolic disorders, is characterized by high blood glucose levels. People living with diabetes continue to increase rapidly all over the world, from 108 million in 1980 to 463 million in 2019, and it is estimated that this number will rise to 700 million by 2045 (24, 25). Microvascular damage is one of the severe complications of persistently high blood glucose levels, which involves several organs including the heart, eyes, kidneys, and even the nervous system. Approximately 40% of diabetic patients will develop diabetic kidney disease (DKD) (26), mainly manifested by glomerular hyperfiltration, progressive albuminuria, declining glomerular filtration rate (GFR), and eventually end stage renal disease (ESRD). Originally, metabolic and hemodynamic factors were thought to be the main causes of renal damage (27), while recently, there is increasing evidence of the involvement of immune system in the development and progression of DKD (28–31). The complement system, an important component of immune system, has also been shown to be engaged in this disease.

Proteomic analysis of laser capture microdissected glomeruli confirmed that C3 and the membrane attack complex (MAC, C5b-9) showed increased in patients with DKD (32). Immunohistochemical staining also revealed high expression of complement factor B, C3d, C5aR, and MAC (33). What is more, it is reported that the urinary excretion of C3b, Bb, and MAC are increased in DKD patients and is demonstrated that the presence of complement split products in the urine is associated with accelerated ESRD and death (34, 35).

It is now believed that increased glycation of proteins, which activates the lectin pathway, and the dysfunction of complement regulatory proteins led by hyperglycemia are the main mechanisms to implicate complement in the development of DKD. *In vitro* experiments have demonstrated that glycation product fructoselysine, whose structure is analogous to mannose, may act as a ligand for MBL and bind to it, initiating complement activation (36). Animal models of DM have also affirmed the role complement lectin pathway played in disease pathogenesis. In the streptozotocininducedtype 1 diabetes mellitus (TIDM) models, mannose-binding lectin (MBL) levels increase (37) and are paralleled with increasing plasma glucose (38). The estimated half-life of recombinant human MBL injected intravenously into diabetic mice was also significantly prolonged (38), suggesting that the elevated MBL in the diabetic models may be due to a combination of increased MBL production and decreased catabolism. Compared to controls, MBL-knockout mice induced by streptozotocin attenuate glomerular hypertrophy, urinary albumin excretion, and renal fibrosis (39). But ficolins, pattern-recognition molecules (PRMs) that cancombine with MASPs to trigger the lectin pathway, may not have a role in the pathogenesis of DKD (40). In type 2 diabetes mellitus (T2DM) rats, the expression of MASP2, a key factor to activate the lectin pathway, is upregulated in renal tubular cells (41). These experimental studies need to be validated in clinical DM patients. In several clinical trials involving patients with T1DM or T2DM, it was confirmed that serum MBL levels were significantly higher in patients with DKD than those DM patients without renal lesions and that high baseline MBL along with CRP levels could be used as a predictor for the development of proteinuria in DM patients (39, 42, 43). And H-ficolin was found to be associated with renal progression, including microalbuminuria, in patients with T1DM (44, 45). Although elevated MASP has been reportedin T1DM (46) and T2DM (47), the potential relevance of MASP and renal damage still requires further investigation in DM patients.

CD59 is a key inhibitor of MAC formation, which is universally expressed in cells. Hyperglycemia induces the dysfunction or inactivation of CD59 after glycation, which proposed the deposition of MAC in renal parenchyma, thereby activating pathways of intracellular signaling, and in turn proinflammatory cytokines and growth factors are released (48). A recent study identified low abundance of urinary CD59 was a significant independent predictor of faster eGFR decline as well as higher risk of progression to ESRD (49).

More components of complement system have also been shown involved in the pathogenesis of DKD. C3a and C5a are well-defined cytokine-like polypeptides that are generated during the activation of the complement system. Li et al. showed that the upregulation of C3a/C3aR and C5a/C5aR was associated with endothelial–myofibroblast transition (EndMT) and fibrosis in glomerular endothelial cells of DKD patients and diabetic rats, and the specific receptor antagonists C3aRA/C5aRA could ameliorate EndMT and renal fibrosis *via* the inhibition of the Wnt/β-catenin pathway both *in vitro* and *in vivo* (50). Decay-accelerating factor (DAF/CD55) is a complement C3 convertase regulator expressed in podocyte. In STZ-induced DKD models, the DAF-deficient mice showed more C3b glomerular deposition and exhibit a more severe disease phenotype and increased histological lesions compared to wild-type mice (51). Additionally, transcriptomic profiling of kidney has also revealed a pivotal role of the C5a/C5aR1 axis in the development of DKD by disrupting mitochondrial agility, which can be restored after inhibiting C5aR (52). What is more, C5aR blockade also alleviated renal dysfunction, ECM deposition, macrophage infiltration, and proinflammatory factor expression in DKD mice, downregulating the expression of many immune response-related genes, such as STAT3 (53). High glucose could also upregulate the expression of factor B and enhance activation of the alternative pathway through mTORC1 activation, which in turn promotes podocyte injury and DKD (33). In clinical trials, plasma levels of C1q, MBL, Bb, C4d, C3a, C5a, and C5b-9 in DKD patients are significantly higher than in diabetes patients without renal involvement (54), and urinary excretion of C3b, Bb, and MAC are also noted (34). A clinical study involved 79 T2DM patients showed that higher concentration of serum C4 level and intensity of glomerular C4c deposits predicted unfavorable renal outcome (55). In a gene-expression analysis of postmortem human kidneys, an upregulation of the expression of C7 in kidney tissue and blood are observed in early DKD, which may be used as a molecular target for detection and/or treatment (56).

**Figure 2:**
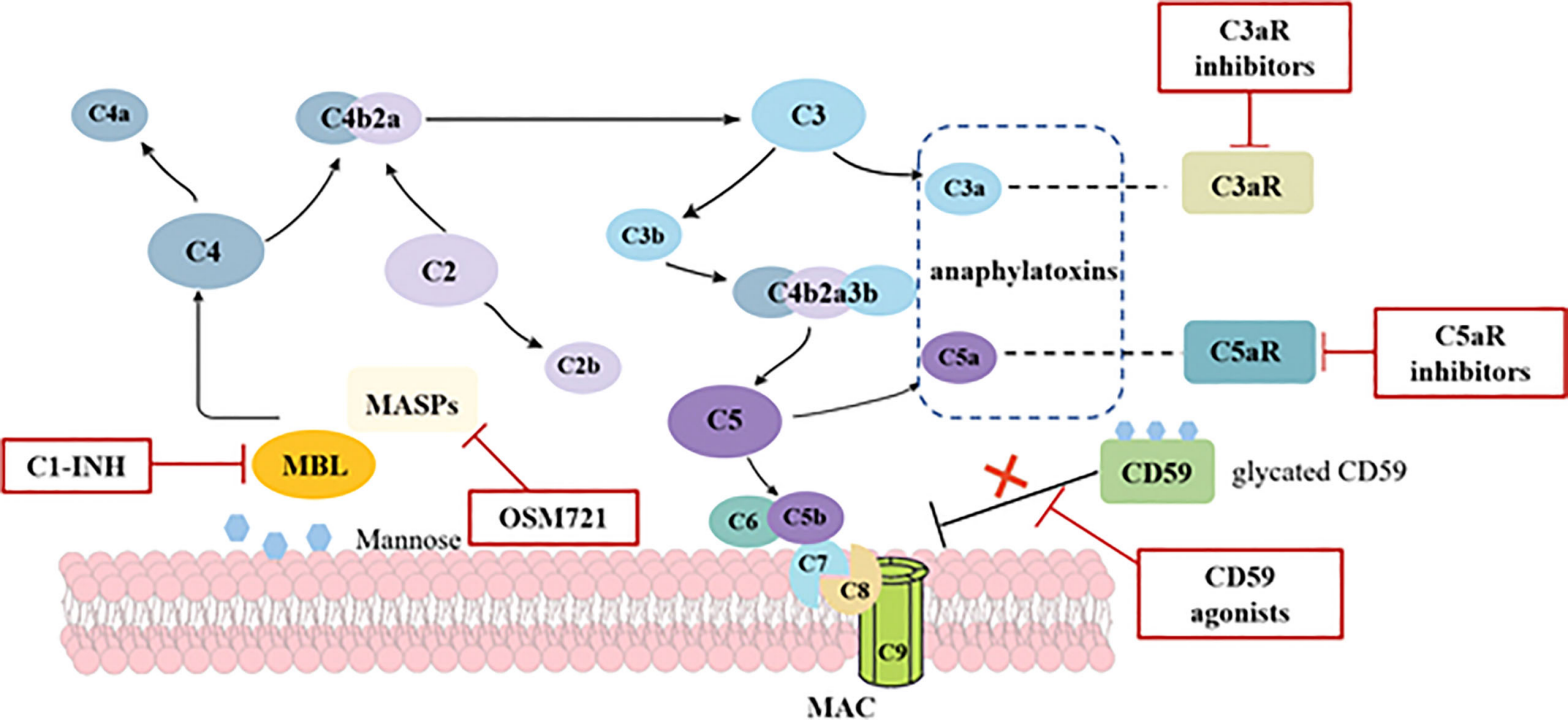
Potential mechanisms and targets of complement activation in diabetic kidney disease. In diabetes, increased glycation of proteins leads to the activation of lectin pathway through MBL and the dysfunction of complement regulatory proteins. Hyperglycemia induces the inactivation of CD59, which is the key inhibitor of MAC formation, thus predisposing to MAC deposition. Anaphylatoxins C3a and C5a are also proved to participate in the pathogenesis of DKD. Potential complement-targeted therapeutics for DKD in red boxes include C1-INH, OSM721, C3aR inhibitors, C5aR inhibitors, and CD59 agonists.

As described above, the activation of complement system is involved in the development of renal damage in DM patients, thereby pathways involving reactive oxidative species (ROS), nuclear factor-κB (NF-κB), and protein kinase C (PKC) are triggered (57). Therefore, complement targeting therapies are gradually becoming a horizon for DKD (**Figure 2**). MBL shares relevant structural and functional homologies with C1q (58); both can be inhibited by C1 esterase inhibitor (C1-INH). As such, C1-INH might be a possible therapeutic approach by suppressing the lectin pathway. Although C1-INH has not been validated in DKD patients, a randomized and placebo-controlled trial indicated patients at a high risk of delayed graft function (DGF) after kidney transplantation required fewer dialysis sessions and had a higher glomerular filtration rate at a 1-year follow-up after treated with C1-INH (59). Another inhibitor of lectin pathway, OMS721, which targets MASP-2, may also provide a new insight about inhibiting the lectin pathway in DKD. Given that the glycation of CD59 leads its inactivation, which enhances complement activity, upregulating the CD59 is regarded as a potential mechanism to inhibit MAC complex formation. Targeting anaphylatoxins C3a and C5a is considered as a promising option, too. Morigi et al. reported that a C3a receptor antagonist improves the podocyte loss, albuminuria, and glomerular injury in T2DM mice (60). Similarly, an inhibitor of the complement cascade (K-76 COONa) also reduced proteinuria and glomerulosclerosis in diabetic rats (61). Although validated in animal models, the safety and efficacy of the inhibitors in humans are still unproven. Eculizumab has proved to be effective in paroxysmal nocturnal hemoglobinuria and atypical hemolytic–uremic syndrome (17). However, considered there is still no data on eculizumab in DKD, more prospective cohort studies are needed.

### Hypertensive Kidney Disease

Hypertension is a major cause of premature death worldwide, with approximately 1.28 billion adults aged 30–79 years worldwide suffering from it, most (two-thirds) living in low- and middle-income countries (62). Nowadays, several large cohort studies have reported that hypertension is an important risk factor for CKD and ESRD (63–65). Hypertensive kidney disease still lacks a clear definition, while it is considered to be the second most common cause of CKD and ESRD, after diabetic kidney disease. Compared to DKD patients, patients with hypertensive kidney disease have less proteinuria, but still accompanied by decreased GFR and increased serum creatinine. Besides oxidative stress in glomerular endothelial cells induced by mechanical injury and activation of RAAS were traditionally known as the main factors of renal damage in hypertension (66), substantial evidence has implicated that several components of the complement system are involved in hypertensive kidney disease (HKD).

We have observed the deposition of complement components in renal biopsy from patients and animals with hypertensive kidney disease in both previous clinical practice and literature reports (67, 68). And clinical trials have shown that serum C3 are paralleled with systolic blood pressure (69, 70). In renal biopsy, studies have found C3c and C5b-9 activated in hypertension-associated TMA, with disordered levels of factor B, D, P, and H while normal C4 level in those patients (71–73). Since C4 is a molecule involved in both classical and lectin pathways, researchers tend to believe that the alternative pathway (AP) is primary in the pathogenetic process of hypertensive kidney disease.

Complement disorders in HKD patients may be a result of multiple conditions. Békássy et al. demonstrated that renin, a kidney-specific enzyme, cleaves C3 into C3a and C3b in a manner identical to the C3 convertase, thus triggering the alternative pathway. And the cleavage can be inhibited by the renin inhibitor aliskiren *in vitro* (74). However, this intriguing possibility still needs to be proven *in vivo* relevance. Factor H is an important negative regulator of AP and can bind to heparin sulfate (HS) in the GBM to protect host cells from complement attack. In patients with hypertensive kidney disease, the GBM is destroyed and exhibits lower HS levels, then the AP would be overactivated (75–77). More importantly, the kidney is a potential complement source (78–81). Tubular epithelial cells can synthesize all complement AP proteins *in vitro*. Glomerular endothelial cells (GECs) also synthesize more CFD and properdin than brain microvascular endothelial cells (BMECs) and human umbilical vein endothelial cells (HUVECs) (82, 83). Hence, the kidney is vulnerable to AP activation because of the altered levels of local complement.

Considering that the complement AP acts as an important role to aggravate kidney tissue damage, targeting the complement system seems to be an optional therapy. Raij et al. reported that DOCA-salt hypertensive C5-sufficient mice showed more severe renal insufficiency and proteinuria than C5-deficient mice and presented more glomerular cell proliferation, cell necrosis, and glomerulosclerosis, extracapillary proliferation morphologically (84). In angiotensin II-induced hypertensive model, C5a receptor 1-deficient mice have lower renal mRNA expression of NGAL and CCL2, as well as less severe albuminuria (85). In spontaneously hypertensive rats (SHR) which show stronger immunohistochemical expression of C3 in glomerulus than controls, after a given inhibitor of C3a receptor, the synthetic phenotype in mesangial cells (MCs) and the production of matrix Gla and collagen IV are suppressed (86, 87). These all remind us that complement inhibition may improve kidney damage caused by hypertension in animal models. Nevertheless, in the Dahl SS rat-fed high-salt diet, after 21 days of treatment with the inhibitor of C3 and C5 convertases CR1, no significant improvement of proteinuria was detected, although C3a production was suppressed (88), which seems to remind us that complement activation in the circulation might not be a critical factor for the kidney damage due to an increased sodium intake caused hypertension. Presently, the use of complement inhibitor in patients with hypertension is limited. A clinical study demonstrated that early treatment with eculizumab can restore renal functionand reduce TMA recurrence in subjects with malignant hypertension (89), while medication targeting C3aR is still not available in the clinic. More research is urgently needed to confirm the feasibility and efficacy of this new treatment.

### Obesity-Related Nephropathy

Obesity is defined as body mass index (BMI) over 30. Statistics from WHO suggest that 650 million adults were obese worldwide in 2016, nearly tripled in 1975. Although obesity has been identified as an independent risk factor for ESRD after adjustment for multiple epidemiologic and clinical features including the presence of diabetes mellitus and hypertension (90), the specific pathogenesis of obesity-related nephropathy is not fully understood. Insulin resistance, inappropriate activation of RAAS, inflammation, and structural changes of kidney are generally regarded as possible explanation (91).

Studies of the complement system in obesity-related nephropathy are limited. Gauvreau et al. reported that mice-deficient in properdin (PKO), which upregulates the alternative pathway by stabilizing the C3bBb complex, had increased body fat mass, as well as a greater excretion of β2 microglobulin and mesangial cell proliferation when fed a high-fat diet compared to controls (92, 93). Reports have also revealed that C3a receptor and C5a receptor contribute to obese adipose tissue inflammationand insulin resistance through macrophage accumulation and M1 polarization (94, 95). Once macrophages are activated, the downstream molecules including the proinflammatory cytokines, chemokines and cellular adhesion molecules are produced, which stimulate subsequently kidney injury and renal fibrosis (96, 97). Lim et al. reported that targeting the receptors of anaphylatoxin C3a and C5a can improve visceral adiposity and inhibit the macrophage signaling, suggesting that it may be a new strategy for treating metabolic dysfunction in animal models (98). Accumulated evidence indicated that serum C3 levels might be a biomarker for insulin resistance in obesity (99) and nonalcoholic fatty liver disease (100). Furthermore, a cross-sectional observational study enrolled 1,191 Chinese adolescents identified that serum C1q was positively related to MS, and may represent a biomarker for predicting obesity or MS in adolescents (101). However, more studies are needed to determine whether complement components can be biomarkers for obesity-related nephropathy.

### Hyperuricemia-Induced Kidney Disease

Uric acid is the end product of purine metabolism, with approximately two-third of urate elimination occurs in kidney (102), whose excessive accumulation leads to hyperuricemia, gouty arthritis, and kidney injury. Uric acid was reported to induce glomerulosclerosis, tubular injury, and interstitial fibrosis, which is suspected to be related with abnormal activation of RAAS and immune system. Although the pathogenesis of hyperuricemia-induced kidney disease is precisely unknown, hyperuricemia has been an independent risk factor for CKD.

Increased urate concentrations result in the deposition of monosodium urate (MSU) crystals in articulations and kidneys, thus leading to structural and functional damage. In a study including 2,731 non-diabetic adults, C3 and C-reactive protein (CRP) was reported to increase positively related with stimulation of uric acid (103). Of note, CRP binds to MSU, thus recruits and activates C1 and MASP1, resulting in the fixation of MAC (104). Additionally, a functional C5 convertase complex assembles at the surface of MSU crystals, leading to the generation of active C5a and C5b (105). C5a then activates the NLRP3 inflammasome in macrophages (106) and promotes the release of IL-1β, which in turn regulates neutrophil recruitment (107), thereby participates in the inflammation caused by hyperuricemia. Nevertheless, there are still no reports about the interaction between renal parenchymal cells and components of the complement system in hyperuricemia. Whether complement system could provide a novel target for hyperuricemia-induced kidney disease needs further investigation.

## Discussion

Various metabolic disorders especially DM and hypertension have become the key factors for the progression of renal damage, which in turn aggravates metabolic disturbances. The mechanisms how complement components interact with kidneys are related to poorly controlled primary diseases, insulin resistance, and chronic inflammation. As we mentioned above, as the most important and fundamental component of innate immune responses, the complement system participates in the metaflammation and tissue damage in kidneys through several pathways (**Table 1**), so that parts of complement components are considered to be novel biomarkers for metabolic kidney diseases. At the same time, therapeutic options targeting the complement system attract the attention of the researchers. Given the important role complement components played in protective immunity against pathogens, long-term blockade of them may lead to potential adverse consequences especially infection. Therefore, more clinical trials are needed to identify the safety and effectiveness of these new inhibitors.

**Table 1:** The role of complement system in the kidney under different metabolic disorders.

Metabolic disorders	Components of complement system	Renal damage
Diabetic kidney disease	The lectin pathway, CD59	Albuminuria, declined eGFR, glomerular hypertrophy, renal fibrosis
Hypertensive kidney disease	The alternative pathway	Glomerular cell proliferation, cell necrosis, glomerulosclerosis, phenotypic transformation of MCs
Obesity-related nephropathy	C3a, C5a, properdin	Albuminuria, MCs proliferation, macrophage accumulation, and polarization
Hyperuricemia-induced kidney disease	The classic pathway The alternative pathway	Albuminuria and renal fibrosis

## Author Contributions

HS conceived and designed the manuscript. LT and ZX did literature searching. ZX drafted the manuscript and drew the figures. HS reviewed and revised the article. All authors contributed to the article and approved the submitted version.

## Funding

This work was supported by Grants from the National Natural Science Foundation of China (82170773, 81873602).

## Conflict of Interest

The authors declare that the research was conducted in the absence of any commercial or financial relationships that could be construed as a potential conflict of interest.

## Publisher’s Note

All claims expressed in this article are solely those of the authors and do not necessarily represent those of their affiliated organizations, or those of the publisher, the editors and the reviewers. Any product that may be evaluated in this article, or claim that may be made by its manufacturer, is not guaranteed or endorsed by the publisher.
